# Probing the Gold Binding Site in Serum Albumin

**DOI:** 10.1155/MBD.1994.522

**Published:** 1994

**Authors:** Rodney E. Sue, Peter J. Sadler, Alan Tucker

**Affiliations:** 1 Department of Chemistry Birkbeck College University of London Gordon House, 29 Gordon Square London WC1H 0PP United Kingdom


					PROBING THE GOLD BINDING SITE IN SERUM ALBUMIN
Rodney E. Sue, Peter J. Sadler and Alan Tucker
Department of Chemistry, Birkbeck College, University of London, Gordon House,
29 Gordon Square, London WC1H 0PP, UK
The antiarthritic drug, aumnofin ("Ridaura') is an orally-active antiarthdtic drug containing
linear Au(I). The gold is co-ordinated by triethylphosphine and the sulfur of tetraacetyl-I-D-1-
thioglucose (HSATg). Deacetylation occurs during absorption and then the thioglucose is displaced
via ligand displacement reactions. Over 80% of the gold in the blood is carded by serum albumin, a
66 kDa single-chain protein with high helical content and three strucumlly similar domains.2
Previous studies have shown that the gold binds to cysteine-34 in domain of albumin, which
has an unusually low pKa (<5).3'4
We have now synthesised a number of potentially antiarthritic gold complexes with a variety of
substitution patterns, including the chiral phosphine complex [PhMeEtPAuCI], I, and studied their
binding to serum albumin by 1H and 31p NMR spectroscopy. The structural changes induced in
albumin by these gold complexes, and comparative reactions with other thiols will be discussed.
We thank the Wellcome Trust, MRC and Wolfson Foundation for support.
References
1. A.E. Finkelstein, D.T. Walz, V. Batista, M. Mizraji, F. Toisman and A. Misher, Ann. Rheum. Dis.,
197e, 3.5, 251.
2. D.C. Carter and X.-M. He, Nature, 1992, 358, 209.
3. O.M. Ni Dhubhghaill, P.J. Sadler and A. Tucker, J. Am. Chem. Soc., 1992, 114, 1118.
4. C.F. Shaw III, Comments Inorg. Chem., 1989, 8, 233.
522

				

